# The hyperbolic effect of density and strength of inter beta-cell coupling on islet bursting: a theoretical investigation

**DOI:** 10.1186/1742-4682-5-17

**Published:** 2008-08-03

**Authors:** Aparna Nittala, Xujing Wang

**Affiliations:** 1 Max McGee National Research Center for Juvenile Diabetes & Human and Molecular Genetics Center, Medical College of Wisconsin and Children's Research Institute of the Children's Hospital of Wisconsin, Milwaukee, WI 53226, USA

## Abstract

**Background:**

Insulin, the principal regulating hormone of blood glucose, is released through the bursting of the pancreatic islets. Increasing evidence indicates the importance of islet morphostructure in its function, and the need of a quantitative investigation. Recently we have studied this problem from the perspective of islet bursting of insulin, utilizing a new 3D hexagonal closest packing (HCP) model of islet structure that we have developed. Quantitative non-linear dependence of islet function on its structure was found. In this study, we further investigate two key structural measures: the number of neighboring cells that each *β*-cell is coupled to, *n*_c_, and the coupling strength, *g*_c_.

**Results:**

*β*-cell clusters of different sizes with number of *β*-cells *n*_*β *_ranging from 1–343, *n*_c _from 0–12, and *g*_c _from 0–1000 pS, were simulated. Three functional measures of islet bursting characteristics – fraction of bursting *β*-cells *f*_b_, synchronization index *λ*, and bursting period *T*_b_, were quantified. The results revealed a hyperbolic dependence on the combined effect of *n*_c _and *g*_c_. From this we propose to define a dimensionless cluster coupling index or CCI, as a composite measure for islet morphostructural integrity. We show that the robustness of islet oscillatory bursting depends on CCI, with all three functional measures *f*_b_, *λ *and *T*_b _increasing monotonically with CCI when it is small, and plateau around CCI = 1.

**Conclusion:**

CCI is a good islet function predictor. It has the potential of linking islet structure and function, and providing insight to identify therapeutic targets for the preservation and restoration of islet *β*-cell mass and function.

## Background

Insulin, secreted by pancreatic islet *β*-cells, is the principal regulating hormone of glucose metabolism. In humans, plasma insulin exhibits oscillatory characteristics across several time scales independent of changes in plasma glucose [1-4]. These oscillations are caused by pulsatile insulin secretion [5,6]. Loss of insulin pulsatility is observed in patients of both type 1 diabetes (T1D) and type 2 diabetes (T2D) [5,7,8], and in relatives with mild glucose intolerance or in individuals at risk for diabetes [9-12]. However, the role of insulin pulsatility in glucose metabolic control and diabetes is still not well understood.

The pulsatile insulin release is driven by the electrical burst of *β*-cell membrane. Theoretically single isolated *β*-cells can burst, and can be induced *in vitro *to release insulin under tightly controlled conditions. But due to the extensive heterogeneity among individual *β*-cells, not all cells will respond to glucose, and for those that do respond, the amplitude, duration and frequency of oscillations are variable [3,13]. In contrast, in *β*-cell clusters or islets where the cell-cell communication is intact, all cells respond to glucose with regular and synchronized oscillations [3,13,14].

Inter-*β *cell coupling is mediated through the gap junction channels formed between adjacent *β*-cells. Gap junctions are specific membrane structures consisting of aggregates of intercellular channels that enable the direct exchange of ions. Such channels result from the association of two hemichannels, named connexons, each contributed separately by the two adjacent cells. Each connexon is an assembly of six transmembrane connexins, encoded by a family of genes with more than 20 members. Using rodent models it was found that connexin36 (Cx36) is the only connexin isoform expressed in *β*-cells [15-18]. Recent study found that Cx36 is also expressed in human islets [19]. Cx36 gap junctions have weak voltage sensitivity and small unitary conductance [20]. This unique combination of properties makes them well suited as electrical coupler, which is important for the regulation of insulin release from *β*-cells [17].

The critical functional role of the gap junctional coupling between *β*-cells has been demonstrated in many experiments. Studies on pancreatic islets and acinar cells revealed that cell-to-cell communication is required for proper biosynthesis, storage and release of insulin, and were nicely reviewed in [21,22]. Single uncoupled *β*-cells show a poor expression of the insulin gene, release low amounts of the hormone, and barely increase function after stimulation [23-25]. Alterations in Cx36 level are associated with impaired secretory response to glucose [15,17,26,27]. Lack of Cx36 results in loss of *β*-cell synchronization, loss of pulsatile insulin release, and significantly higher basal insulin release in the presence of sub-stimulatory glucose concentration from isolated islets [28]. Blockage of gap junctions between *β*-cells also similarly abolish their normal secretory response to glucose [3,25,29]. Restoration of *β*-cell contacts is paralleled by a rapid improvement of both insulin biosynthesis and release [23-25]. Further support for this concept comes from the finding that a number of tumoral and transformed cell lines that do not express connexins show abnormal secretory characteristics [30]. Transfection of the cells with a connexin gene corrected the coupling and some of the secretory defects [30]. In addition to the functional role in insulin secretion, study with transgenic mice overexpressing Cx36 showed that it protects *β*-cells against streptozotocin (STZ) and cytokine (IL-1*β*) damage, and loss of the protein sensitizes *β*-cells to such damages [22]. On the other hand, impaired glucose tolerance can compromise the gap junctional channels. In vitro study of freshly isolated rat islets has found that short exposure (30 min) to glucose can modify gap junction configuration [31] whilst a chronic increase in glucose decreases Cx36 expression [32], suggesting that compromise of *β*-cell coupling may be implicated in the early glucotoxicity and desensitization phenomena, and may therefore be relevant to diabetes pathophysiology.

Theoretical models were developed to describe the *β*-cell oscillation [33-38], which also revealed how an increased regularity of glucose-dependent oscillatory events was achieved in clusters as compared to isolated islet *β*-cells [35-38]. Together, these experimental and modeling results strongly indicate the essential role of cell-cell communication in normal *β*-cell function, which may account for the hierarchical organization of *β*-cell mass. The insulin secreting *β*-cells, together with the other endocrine cells, comprise only about 1–2% of the total pancreatic mass. Rather than being distributed evenly throughout the pancreas, they reside in a highly organized micro-organ, the pancreatic islet, with specific 3D morphostructure, copious intercellular coupling and interactions, and are governed by sensitive autocrine and paracrine regulations. This organization, not individual *β*-cells, is the basis for generating the insulin oscillation and a proper glucose dose response. Therefore one would expect that the morphostructural integrity of islets, namely, the interactions and the three-dimensional architecture among various cell populations in islets, is critical for islet function. Indeed, in islet transplantation studies it has been found that these characteristics are predictive of *in vivo *function and survival of islets, as well as the clinical outcome after transplantation [39]. Despite the many published models of pulsatile insulin release, a quantitative investigation of the functional role of islet *β*-cell's cytoarchitectural organization was not available until recently [40].

In our previous work we have proposed that a *β*-cell cluster can be described by three key architectural parameters: number of *β*-cells in the cluster *n*_*β*_, number of neighboring *β*-cells that each *β*-cell is coupled with *n*_c_, and intercellular coupling strength *g*_c _[40]. Traditional islet simulation has assumed a simple cubic packing (SCP) arrangement of *β*-cells, with 6 nearest neighbors for each cell, i.e. *n*_*c*,max _= 6. We found that this model significantly underestimates the neighboring cells each *β*-cell has, with which potential intercellular coupling could be formed [40]. It is therefore limiting to investigate the effect of varying proportions of non-*β *cells (which do not couple with *β*-cells), or the functional consequence of architectural perturbations such as compromised degree of intercellular coupling resulting from *β*-cell death. We therefore introduced a new hexagonal closest packing (HCP) model with 12 nearest neighbors for each cell, and *n*_*c*,max _= 12. It provides a much more accurate approximation to the cytoarchitectural organization of cells in islet tissue. Experimental studies of islet *β*-cell clusters also implicated a hexagonal organization of cells [41,42] (see figure 7 on page S15 of [41], figure 5 on page 40 of [42], for example). Further, it was estimated that in rodent islets about 70% of the cells are *β*-cells; this corresponds to an effective *n*_c _~ 8.4 (as 30% of the 12 nearest neighbors are non-*β *cells) in our HCP model, which is consistent with laboratory measurements of the degree of inter-*β *cell coupling [43]. Human islets are believed to contain proportionally much less *β*-cells, at ~50% [44,45], which corresponds to *n*_c _~ 6.

Using this new *β*-cell packing model, we examined, for the first time, the functional dependence of islet oscillation on its architecture. Optimal values of *n*_*β*_, *n*_c _and *g*_c _at which functional gain is maximized are obtained [40]. In this study, we further investigate islet-bursting phenomenon as reflected in three functional measures: fraction of *β*-cells that could burst *f*_b_, synchronization index *λ*, and bursting period *T*_b_. We will specifically examine the influence of structural perturbation to *n*_c _and *g*_c_, and if a composite measure of islet morphostructural integrity can be defined from them. As in previous study, we focus the investigation from the perspective of high frequency oscillation resulting from the feedback loops of intracellular calcium currents, which is in the time scale of ~10–60 sec. We reserve the more comprehensive investigation of *β*-cell oscillation at different time scales in future work.

## Results

### Sorting cells using Lomb-Scargle periodogram

The first step post simulation of a *β*-cell cluster is to determine the bursting status of each *β*-cell in the cluster. In general it can be a burster, a spiker, or a silent cell [40]. A burster is defined as a cell capable of producing a sequence of well-defined regular bursts which correlate with the period between consecutive peaks and nadirs in the calcium signal or membrane action potential. In contrast, a spiker usually produces uncontrolled continuous voltage spikes and does not spend any significant time in the plateau phase of sustained oscillation, thereby being unable to generate a glucose dose response. A silent cell is one which remains in the hyperpolarized state throughout, and thus remains inactive in the insulin secretion process. In our previous work, we used an empirical rule based on the peak and nadir information of the *s*(*t*) signal (the slow variable of the potassium channel, see equations 4–5 in methods) to distinguish between spikers and bursters. In this study we introduce a more analytical method. The sorting hat (Rowling J.K.) we utilized is the Lomb-Scargle periodogram [46,47], which describes power concentrated at particular frequencies. We applied it to intracellular calcium concentration [*Ca*(*t*)].

**Figure 1:**
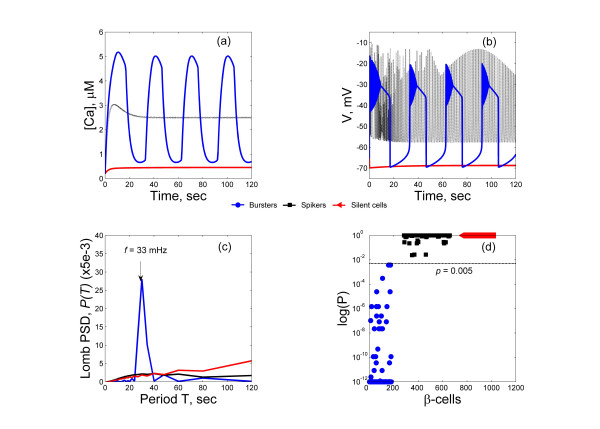
Cell sorting using Lomb-Scargle periodogram. (a) Calcium profiles, (b) membrane action potential profiles, and (c) Lomb-Scargle periodogram, of a burster cell, a spiker cell and a silent cell. The burster has a clear peak frequency at *f *= 33 mHz (0.033 sec^-1^), whereas the spiker and silent cells have broad spectra. (d) Distribution of the principal peak *p *values. All cells with *p *< 10^-12 ^were plotted at *p *= 10^-12^. The burster cells form a distinct group from others, with *p *< 0.005 (dashed black line).

Figure 1 presents the calcium and membrane voltage profiles of three sample cells – a burster, a spiker and a silent cell, along with their computed Lomb-Scargle periodograms. As we can see, the spiker and the silent *β*-cells have a broad frequency spectrum and power is spread out over a wide-range of frequencies, whereas for the burster *β*-cell, the distribution is much narrower and the major peak frequency was observed at 33 mHz. The *p*-value of the principal frequency component of the burster cell assumes a significantly low value with *p *< 10^-12^, while it is >0.4 for the spiker and silent cells. In this study the threshold *p*-value for burster cell is set to be 0.005. We find that this algorithm distinguishes well the burster cells from the rest. Figure 1d presents the distribution of *p*-values for 819 *β*-cells from three *β*-cell clusters: a HCP-323, a SCP-343, and a HCP-153 cluster. Cells with regular bursting clearly segregate from others into a distinct group. Spikers with a very regular spiking frequency can also have marginally significant principal peaks, but normally with *p *> 0.05. The algorithm was tested extensively and zero misclassification was found for all the clusters we have simulated. Hence we believe that the *f*_b _estimation using the Lomb-Scargle periodogram is accurate.

### The hyperbolic relationship between *g*_c _and *n*_c_, and the cluster coupling index CCI

To investigate the functional role of islet structure characterized by (*n*_*β*_, *n*_c_, *g*_c_), we simulated for over 800 different structural states of islet (see figure 5 in methods). Our previous study has revealed a quantitative dependence of islet function on the 3D morphostructural organization of its *β*-cells. This raises the question if a composite measure of islet architectural integrity can be defined to capture the dependence and to develop predictive models of islet function. Given a *β*-cell cluster, the architecture intactness of the whole cluster depends critically on both the individual pair-wise cell coupling strength (*g*_c_) and the number of couplings each *β*-cell has (*n*_c_).

Specifically, the coupling term in equation 3 (see methods) can be written as:

(1) $$\sum_{j=\text{all cells coupled to }i}g_{\text{c}}\left(V_{i}-V_{j}\right)=\left(n_{\text{c}}\cdot g_{\text{c}}\right)\times \left(V_{i}-{\bar{V}}_{i}\right)$$

where ${\bar{V}}_{i}=\frac{1}{n_{c}}\sum_{j=\text{all cells coupled to }i}V_{j}$ is the mean field value of all the nearest neighbors of cell *i*. This suggests that mean (*n*_c_·*g*_c_) can be a measure that describes the coupling integrity of the islet.

For a normal islet, the distribution of (*n*_c_·*g*_c_) is around a constant.

**Figure 2:**
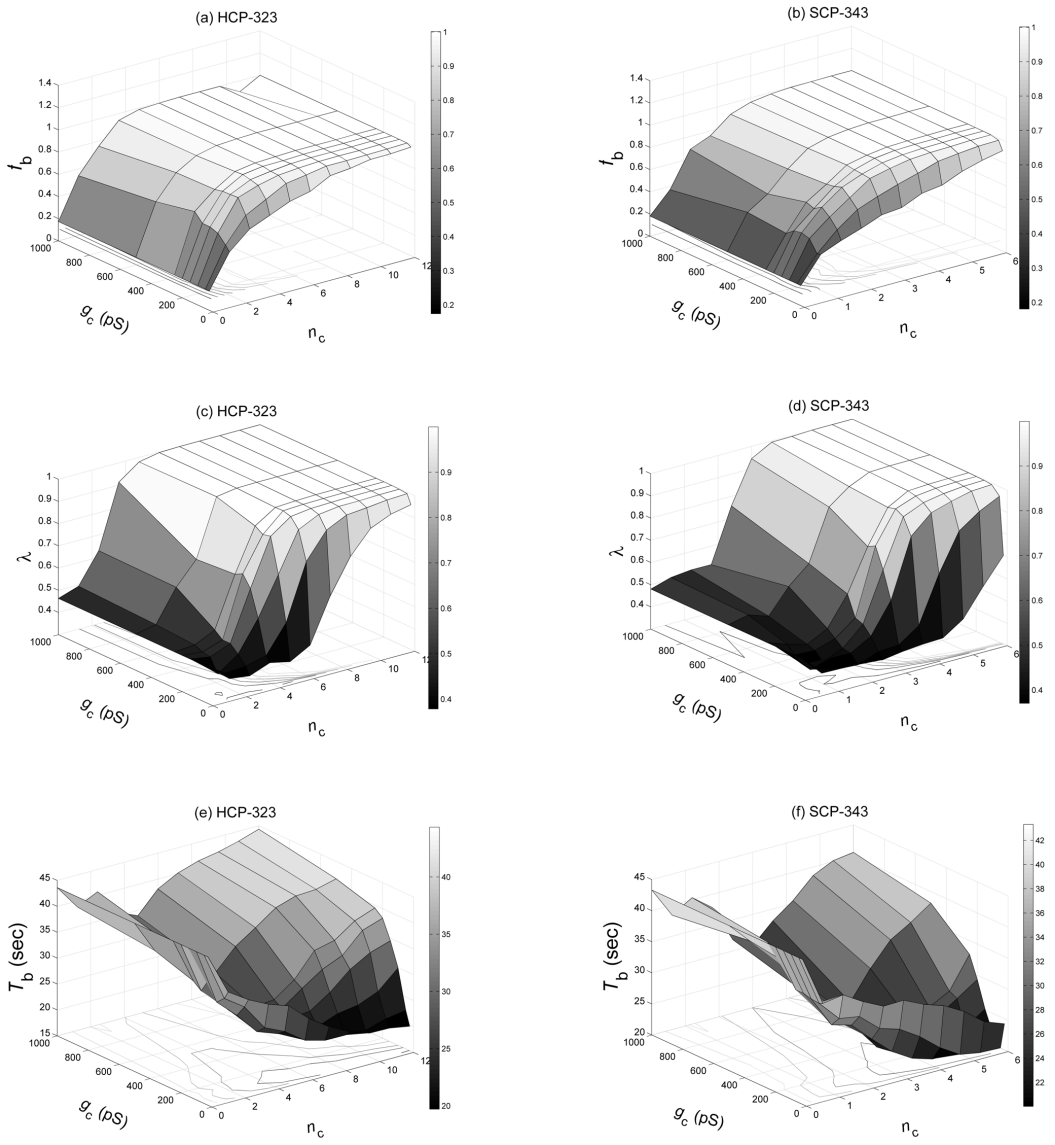
Fraction of burster cells *f*_b_, synchronization index *λ*, and bursting period *T*_b _plotted for a HCP-323 *β*-cell cluster (a, c and e) and a SCP-343 cluster (b, d, and f) on the *g*_c_-*n*_c _plane. A clear hyperbolic relation is visible.

We have evaluated the three functional measures *f*_b_, *λ*, and *T*_b _for all *β*-cell clusters that we have simulated. Figure 2 presents the results for the HCP-323 and SCP-343 clusters on a *g*_c_-*n*_c _plane. It is of interest to note that they indeed follow a hyperbolic response to *g*_c _and *n*_c _at lower values of *g*_c _or *n*_c_, and plateau at higher values. Other clusters with different *n*_*β *_emulate these responses.

The islet cell coupling and cytoarchitecture are likely compromised during the onset and progression of diabetes. During prediabetic development of disease, as well as after diabetes onset, significant loss of *β*-cell mass occurs [48,49]. This will reduce the number of available *β*-cells for coupling, thus reducing the value of *n*_c_. During T1D specifically, the infiltrating immune cells will further reduce *n*_c_, as many neighboring cells would be replaced by the immune cells. Though the role of gap junction conductance in human diabetes has not been investigated in depth, animal model studies have indicated its potential involvement in both T1D and T2D [22]. The gap junction conductance *g*_c _between each pair of cells is the product of number of gap junctional channels formed between them and the specific conductance of each channel, with the latter depending on the channel configuration among other factors. Using transgenic rodent models, it has been shown that the amount of gap junctions directly affects the cell-cell communication and the synchronization of *β*-cell oscillation [28,50]. Reduced amount of gap junctions leads to loss of regular oscillation and the pulsatile insulin release at stimulatory levels of glucose, and increased insulin output at basal glucose. These characteristics of pancreatic dysfunctions mimic those observed in diabetes, and are suggestive of a role of gap junction in the pathophysiology of diabetes [22]. Conversely, gap junctions are dynamic structures, their number, size, and configurations are readily affected (regulated) by environmental conditions, including the glucose level [31,32]. Therefore diabetes progression likely can also affect the value of *g*_c_.

Bearing in mind the significance of the combined effect of *g*_c _and *n*_c _in determining cluster coupling, and their potential importance in the pathological development of disease, we propose a dimensionless cluster coupling index:

(2) *CCI *= (*n*_c_·*g*_c_)/*C*_0_

as an islet cytoarchitectural integrity descriptor, where *C*_0 _= (*n*_c,0_·*g*_c,0_) is a normalization constant, and *n*_c,0 _and *g*_c,0 _are their corresponding normal physiological values. In normal rodent islets, ~70% of the islet cells are *β*-cells, which gives *n*_c,0 _~ 8.4 assuming hexagonal arrangement. The gap junctional conductance has been measured, and found to distribute around *g*_c,0 _~200 pS [51,52]. Therefore *C*_0 _~ 1680 pS•cell. Less is known about human islets except that the proportion of *β*-cells is smaller, at ~50% [44,45], which gives *n*_c,0 _~ 6.0. The *g*_c,0 _value of human islets is still to be measured. It would of interest to examine if human islets have higher *g*_c,0 _(most likely by forming more gap junction channels between pairs of neighboring *β*-cells) compared to rodent islets, to compensate for the smaller *n*_c,0 _value.

**Figure 3:**
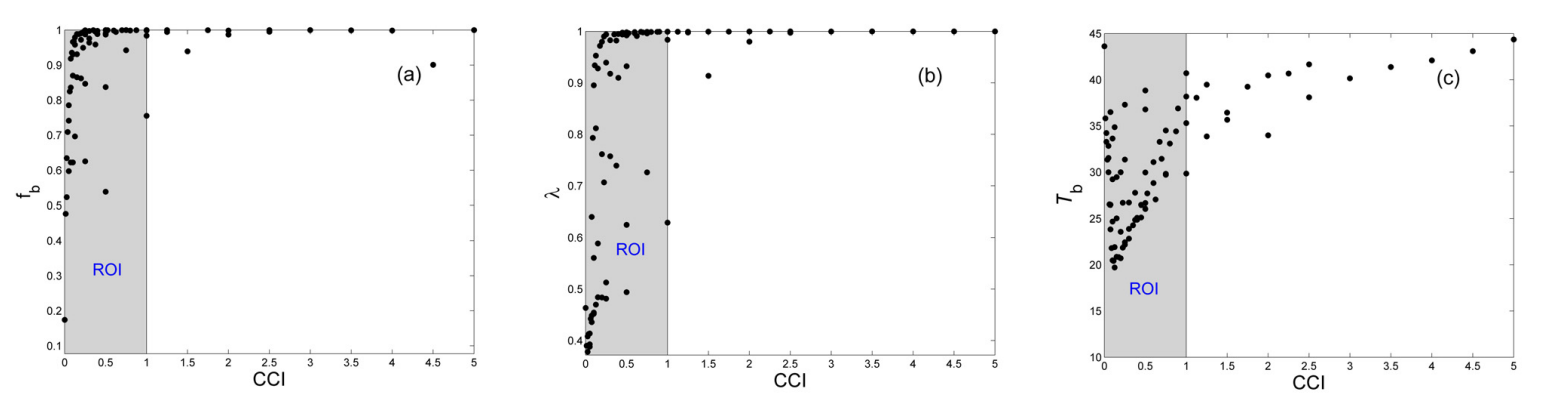
Islet functional measures versus CCI exhibiting potential ROI for therapy (shaded areas). (a) Fraction of burster cells *f*_b_. (b) Synchronization Index *λ*. (c) Bursting period *T*_b_.

Figure 3 presents the dependence of the three functional measures on CCI for all HCP *β*-cell clusters we simulated, assuming *C*_0 _= 1680 pS•cell. Clearly when CCI<1.0, all three measures increase monotonically with increasing CCI value. Little additional functional gain is obtained in the region of CCI>1.0. Values of CCI greater than 1.0 represent higher states of coupling in the islet network system. Islet is robust in its function with strong inter-communication and synchronization. The functional gain of increasing either *g*_c _or *n*_c _when the other is intact, is not of much therapeutic value. This region is of interest to investigate the uplimit of islet connectivity and how this might have evolved. It would also be of interest to study the CCI values of real islets, their distribution, and the upper limit of islet evolution in terms of developing gap junctions and neighborhood coupling.

During diabetes *n*_c _and *g*_c _values are likely compromised, either contributing to or resulting from problems in glucose tolerance. Reduction either in *n*_c _or *g*_c _will lower the value of CCI. When CCI<1.0, extensive variation in all three measures is evident, indicating functional impairment and instability. For consideration of potential therapeutic treatment, this is the critical region for investigation of mechanisms to restore islet structural integrity and functionality by improving *g*_c _and/or *n*_c_, and bringing CCI back to its desired value. For this reason we denote CCI<1.0 as the region of interest (ROI) for potential therapy (shaded areas in figure 3).

## Discussion

Previously we have, for the first time, studied the functional dependence of islet pulsatile insulin release on its cytoarchitectural organization of *β*-cells [40]. In the current study, we further investigated two key islet structural parameters *g*_c _and *n*_c _on islet bursting properties, which are likely involved in the pathophysiology of diabetes. Although numerous experiments have demonstrated the importance in islet function of cell-cell communication between *β*-cells mediated through the gap junction channels, few studies have examined quantitatively the functional role of density and strength of the gap junctions. As synchronization of *β*-cells in their electrical burst and insulin release is the hallmark of normal islet function, we focused on three related functional measures: fraction of *β*-cells that can burst *f*_b_, synchronization index *λ*, and bursting period *T*_b_. We specifically examined the hyperbolic response of *β*-cell cluster function to the combined input of *g*_c _and *n*_c_. This means islet functionality can be preserved by manipulating any one or both of them. For example under weak *g*_c _caused by low expression of gap junction proteins (Cx36), increasing the value of *n*_c _will result in improved number of burster cells, bursting pattern and synchronization, and improved islet function. Similarly, when infiltration of immune cells and *β*-cell loss leave few well-connected neighboring *β*-cells (reduced *n*_c_), targeting the gap junction strength (improving *g*_c_) of existing couplings can improve the bursting and synchronization.

We characterized the hyperbolic effect of *g*_c _and *n*_c _on islet function in a dimensionless composite measure CCI. We showed that this measure correlates well with islet functional performance. We believe that CCI has the potential to be an index of islet's well-being that is predictive of islet function, and thus a key factor linking structure and function. It can provide insight to the intrinsic compensation mechanism of islet cells when damage occurs. The complexity of islet function can be better understood when associating it with CCI.

Human islet biology is difficult due to tissue inaccessibility. Most of our current knowledge is obtained and extrapolated from animal studies. However, recent studies revealed cytoarchitectural differences between human and animal islets [44,45]. Specifically, in the frequently used rodent models, an islet contains significantly lower proportions of non-*β *cells compared to in humans, ~30% versus ~50% (this gives, on average, *n*_c _~ 8.4 versus *n*_c _~ 6, in our HCP cell cluster model). It was further estimated that about 70% of *β*-cells exclusively associate with *β*-cells in rodent islets (namely 70% *β*-cells have *n*_c _~ 12), whilst in human islets, this number can be as low as 30% (only 30% *β*-cells have *n*_c _~ 12) [44,45]. These reports suggest that rodent islets may have much higher *n*_c _than human ones. The functional implication of such architectural difference is still not known, but clearly cannot be extrapolated linearly. We believe that our work, aimed at achieving a quantitative understanding of islet function and cytoarchitecture, will help us to study human islet biology utilizing animal models. For example, it will also be of interest to examine if CCI is conserved across species, and if it can serve as a scale-invariant index that unveils a common reigning principle across species of islet functional dependence on structure.

Investigation of islet function and structure is no doubt of interest to the study of glycemic control, diabetes pathogenesis, and the related metabolic syndromes. Such a study is *sine qua non *for understanding pathological progression of *β*-cell mass and function loss, and islet tissue engineering and transplantation, to name a few [39,40]. Under many physiological/pathological conditions, such as pregnancy, puberty, and diabetes, *β*-cell mass is modified. Often the modification is more profound than a mere change of islet size or islet number. For example in T1D the infiltrating immune cells spread from peripheral islet vessels to the centre of a given islet, causing *β*-cell apoptosis across the islet [53] and modification of islet architecture in addition to its total *β*-cell mass. To many with T1D, islet transplantation represents a viable hope to control hyperglycemia; however, significant loss of islet mass and function are observed both short term and long term after transplantation [54]. It is still not clear what exactly the transplanted islets go through. Predictive models of islet function and survival post transplantation are much needed. Several commonly used parameters in islet preparation quality control: islet size (*n*_*β*_), percent of cells that are *β*-cells (affects *n*_c_), non-apoptotic *β*-cells (affects both *n*_c _and *g*_c_), etc [39], actually constitute the structural framework of the islet. Very recently, it has been explicitly pointed out that the morphostructural integrity of the islets is critical and predictive of *in vivo *function and clinical outcome in islet allotransplantation, and should be studied more [39]. We believe our study provides a starting point for better understanding these issues.

In this study, we focused the investigation on islet architectural measures, and how they affect islet oscillation. For simplicity, as in previous study, we adopted an oscillation model that describes only the high frequency (at the time scale of ~10–60 sec) component resulting from the feedback loops of the intracellular calcium currents. To have a more comprehensive physical description and better understanding of the pulsatile insulin secretion from islets, and how it depends on islet cytoarchitecture, the other components, especially the intracellular metabolism and the signal transduction pathway of glucose induced insulin release need to be included: the oscillation of glycolysis, ATP/ADP ratio, cAMP, and the other metabolic factors such as NADPH, glutamate, glutamine; the cytosolic calcium, and the exchange of calcium with ER and the effect of ER stress; etc [55-66]. These coupled with the electrical current oscillation, would generate an additional slow rhythm at the time scale of 2–10 min. The latter is important as it is at a more readily measurable time scale with available laboratory techniques. It would be of interest to investigate how the intracellular pathways and intercellular connections are coupled in determining the islet function, how the properties of individual *β*-cells affect the islet function through the network of coupled *β*-cells, and whether in a coupled network, the islet is more robust to defects in individual *β*-cells such as problems in the intracellular pathways. In this sense, our work only represents the first step towards developing practical models and quantitative measures of islet architecture and investigating its role in islet function. More sophisticated models and laboratory studies are needed. The electrophysiology of islet and *β*-cell oscillation, and evaluation of islet architectural organization, are all experimentally challenging. We believe that such theoretical analysis, though may only represent an initial minimal model approach, are meaningful to gain some insight, and to help design the most relevant and feasible experiment to examine the key factors in these issues.

## Methods

### Mathematical model of the electrical excitability of *β*-cells

As we have previously described in [40], we adopt the formulation developed by Sherman et al [67,68] of the Hodgkin-Huxley model for *β*-cell electrical excitability, for its simplicity:

(3) $$\begin{array}{c} C_{m,i}\frac{dV_{i}}{dt}=-(I_{Ca,i}+I_{K_{\text{ATP}},i}+I_{K,i}+I_{S,i})-.....\\ \sum_{j=\text{all cells coupled to }i}g_{\text{c}}\left(V_{i}-V_{j}\right) \end{array}$$

The ionic current terms include the fast voltage-dependent L-type Ca^2+^-channel current *I*_Ca_, the glucose sensitive K_ATP _channel current *I*_*KATP*_, the voltage-dependent delayed rectifier K^+ ^current *I*_*K*_, and a slow inhibitory K^+ ^current *I*_*S*_, given by:

(4) $$\begin{array}{l} I_{K_{\text{ATP}}}=g_{K_{\text{ATP}}}O_{K_{\text{ATP}}}(V-V_{K})\\ I_{Ca}=g_{Ca}\cdot m_{\infty }\left(V-V_{Ca}\right)\\ I_{K}=g_{K}\cdot n\left(V-V_{K}\right)\\ I_{S}=g_{S}\cdot s\left(V-V_{K}\right) \end{array}$$

where *g*_*KATP*_, *g*_*Ca*_, *g*_*K*_, *g*_*S *_are channel conductance. The activation parameters *n*, *s *are given by

(5) $$\begin{array}{l} \frac{dn}{dt}=\frac{1}{\tau_{n}}\left(n_{\infty }-n\right)\\ \frac{ds}{dt}=\frac{1}{\tau_{s}}\left(s_{\infty }-s\right) \end{array}$$

with $m_{\infty }=\frac{1}{1+\exp\left(\left(V_{m}-V\right)/\theta_{m}\right)}$, $n_{\infty }=\frac{1}{1+\exp\left(\left(V_{n}-V\right)/\theta_{n}\right)}$, $s_{\infty }=\frac{1}{1+\exp\left(\left(V_{s}-V\right)/\theta_{s}\right)}$ being the fraction of open channels for the corresponding currents respectively at steady state. The parameters *V*_*m*_, *V*_*n*_, *V*_*s*_, and *θ*_*m*_, *θ*_*n*_, *θ*_*s *_are constants that describe the dependence of channel activation on membrane voltage V. The change in intercellular calcium concentration is given by

(6) $$\frac{d{\left[Ca^{2+}\right]}_{i}}{dt}=f\left(-\alpha_{i}I_{Ca,i}-k_{Ca,i}{\left[Ca^{2+}\right]}_{i}\right)$$

where *f *is the fraction of free Ca^2+ ^and *k*_Ca _is the removal rate of Ca^2+ ^in the intracellular space. *α *is a conversion factor from chemical gradient to electrical gradient. For a more detailed explanation of the model equations, parameters and their values, and the implementation, please refer to [40]. The numerical simulation was performed for the 4 ODEs given in equations 3, 5, and 6.

### The HCP model of *β*-cell cluster

We have previously introduced the HCP model of islet cytoarchitecture to simulate the functional consequence of varying structure [40]. In this model each cell has 6 nearest neighbors in 2D (*n*_*c*,max _= 6), and 12 in 3D (*n*_*c*,max _= 12). Setting up the simulation for HCP *β*-cell clusters is more intricate than the SCP model, and we have developed a cell labeling algorithm [40]. Briefly, given a *β*-cell cluster with edge size *n*, labeling of cells starts with the center or the primary layer. It is a 2D regular hexagon of edge size *n*, with a total of 3*n*^2^-3*n*+1 cells. The remaining *n*-1 layers on each side (top and bottom) of the primary layer, starting from immediate layer adjacent to it, alternate between being an irregular hexagonal (IH, the six sides and internal angles are not all equal) layer, and a regular hexagonal (RH) layer. The edge size decreases each time when traversing up or down. The number of cells in IH and RH layers is given by 3(*r*-1)^2 ^and 3*r*^2^-3*r*+1 respectively where *r *is the edge size of that layer. When *n *is even, a 3D HCP cluster ends with an IH-layer on its surface and when *n *is odd, it ends with an RH-layer on its surface [40]. This definition ensures that our HCP clusters are symmetric along all directions, which simulates the natural growth of pancreatic islets. Lastly, the program generates nearest neighbor list for each *β*-cell based on the Euclidean distance between cells.

All cell j located at (*x*_*j*_, *y*_*j*_, *z*_*j*_) belongs to the neighborhood of cell i at (*x*_*i*_, *y*_*i*_. *z*_*i*_) if the Euclidean distance between the two cells is 1, namely:

(7) $$\text{Nbr}(\text{Cell }i)=\left\{\text{Cell }j|\sqrt{{\left(x_{i}-x_{j}\right)}^{2}+{\left(y_{i}-y_{j}\right)}^{2}+{\left(z_{i}-z_{j}\right)}^{2}}=1\right\}$$

This neighbor list is then utilized to set up the $\sum_{j=\text{all cells coupled to }i}g_{\text{c}}\left(V_{i}-V_{j}\right)$ term in equation 3.

**Figure 4:**
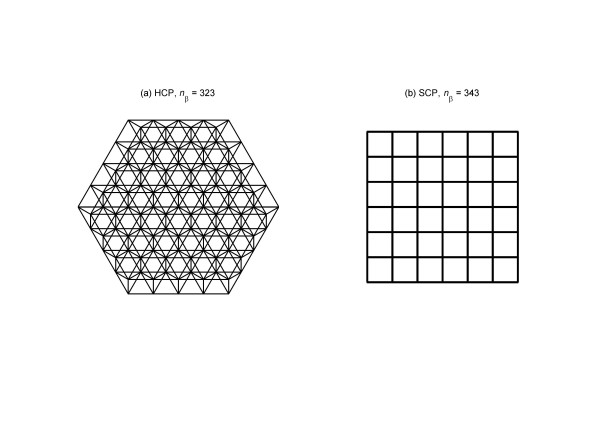
3D HCP and SCP cell clusters projected on a 2-dimensional *x-y *plane. (a) A HCP-323 cluster with edge size 5. Each cell is connected with *n*_c _= 12 neighbors, 6 from the same layer and 6 from the layers above and below. (b) A conventional SCP 7 × 7 × 7 cluster with *n*_c _= 6 for each cell.

Figure 4 presents the top view of a 3D HCP-323 and a SCP-343 cell cluster. Evident from the figure is the complexity of HCP but the added advantage of a higher degree of intercellular coupling, as well as the simplicity of SCP with its limited intercellular coupling.

### Numerical Simulations

**Figure 5:**
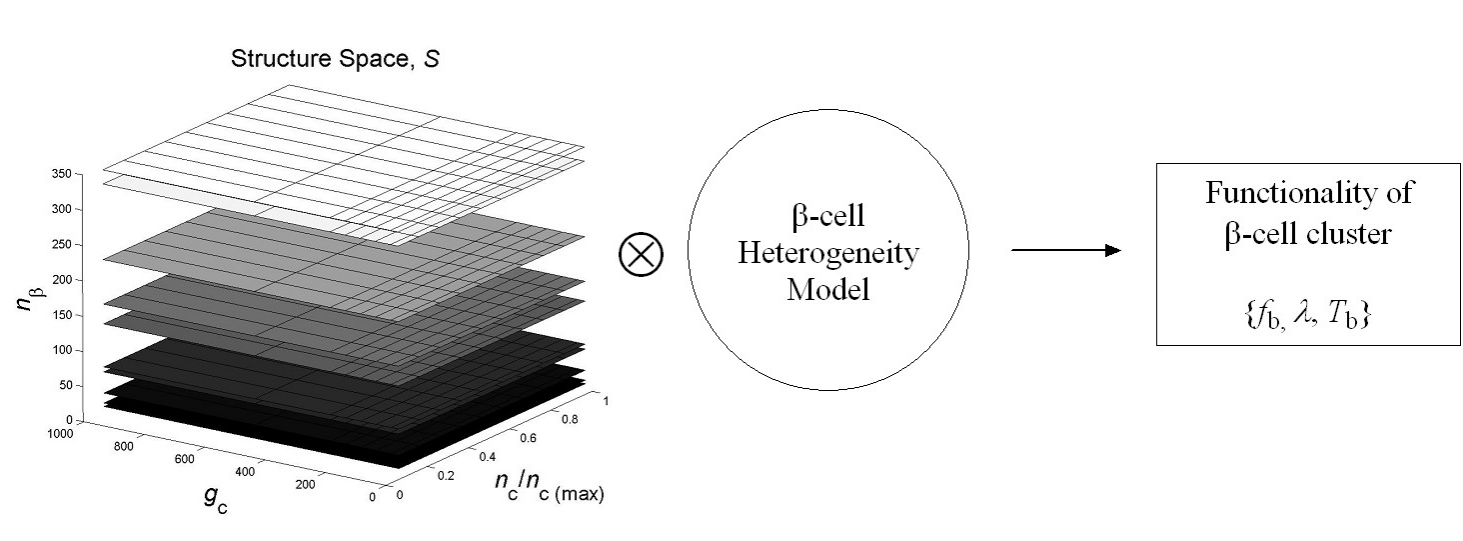
Simulation schema.

HCP and SCP *β*-cell clusters of different sizes with number of *β*-cells *n*_*β *_ranging from 1–343, number of inter *β*-cell couplings of each *β*-cell *n*_c _varying between 0–12, and coupling strength *g*_c _spanning from 0–1000 pS, were simulated, as described in figure 5. Totally we simulated for over 800 different clusters. For each point in the structure space ***S***: (*n*_*β*_, *n*_c_, *g*_c_), 10 replicate clusters were simulated with the biophysical properties of individual *β*-cells following the heterogeneity model as previously described, in table 2 of [40]. 500 uncoupled single *β*-cells were also simulated, which corresponds to point (1, 0, 0) in ***S ***(figure 5). This provides the baseline information for analyzing the functional characteristics of coupled cell clusters.

Simulation for *n*_c _is modulated by randomly decoupling varying percentages of *β*-cells from the rest. This is designed to simulate the loss of *β*-cell mass under pathological conditions, or the presence of non *β*-cells (mainly *α*- and *δ*-cells) in natural islets. It is known the non-*β *islet cells do not synchronize with *β*-cells or among themselves [69], presumably because they do not couple to *β*-cells, and the coupling among themselves are too sparse to coordinate their dynamic activities. Gap conductance *g*_c _is varied from a no coupling state (where each cell is in a quarantine-like state and functioning without any communication, *g*_c _= 0 pS) to a strongly coupled state of 1000 pS.

### The Sorting Hat for *β*-cells

We introduce the Lomb-Scargle periodogram [46,47], which describes power concentrated in a particular frequency, namely, the power spectral density (PSD), to sort the bursting status of *β*-cells. We adopt this method over the more commonly used Fourier method for two reasons: (1) it does not require evenly spaced time series while the Fourier method does. It may not be a major concern if we restrict to only the analysis of the intracellular calcium (figure 1, upleft), and only the steady state solution. But other parameters, particularly the membrane potential, exhibit more complex temporal patterns, with high frequency oscillation overlaying the plateau phase of the slower oscillations (figure 1, upright). (2) the Lomb-Scargle Periodogram comes with a statistical method to evaluate the significance of the observed periodicity [47] while Fourier transform method does not.

Briefly, let *y*_i _be the time-dependent intra-cellular calcium [*Ca*(*t*)] obtained by simulation at each time *t*_*i*_, where *i *= 1,2,..., *N*, with mean $\tilde{y}$ and variance *σ*^2^. The Lomb-Scargle periodogram *P*(*ω*) at an angular frequency of *ω *= 2*πf *is computed according to the following equation:

(8) $$P(\omega )=\frac{1}{2\sigma^{2}}\left\{\frac{{\left[\sum_{i=1}^{N}\left(y_{i}-\tilde{y}\right)\cos\omega \left(t_{i}-\tau \right)\right]}^{2}}{\sum_{i=1}^{N}\cos^{2}\omega \left(t_{i}-\tau \right)}+\frac{{\left[\sum_{i=1}^{N}\left(y_{i}-\tilde{y}\right)\sin\omega \left(t_{i}-\tau \right)\right]}^{2}}{\sum_{i=1}^{N}\sin^{2}\omega \left(t_{i}-\tau \right)}\right\}$$

where the constant *τ *is obtained from:

(9) $$\tan(2\omega \tau )=\frac{\sum_{i=1}^{N}\sin2\omega t_{i}}{\sum_{i=1}^{N}\cos2\omega t_{i}}$$

**Figure 6:**
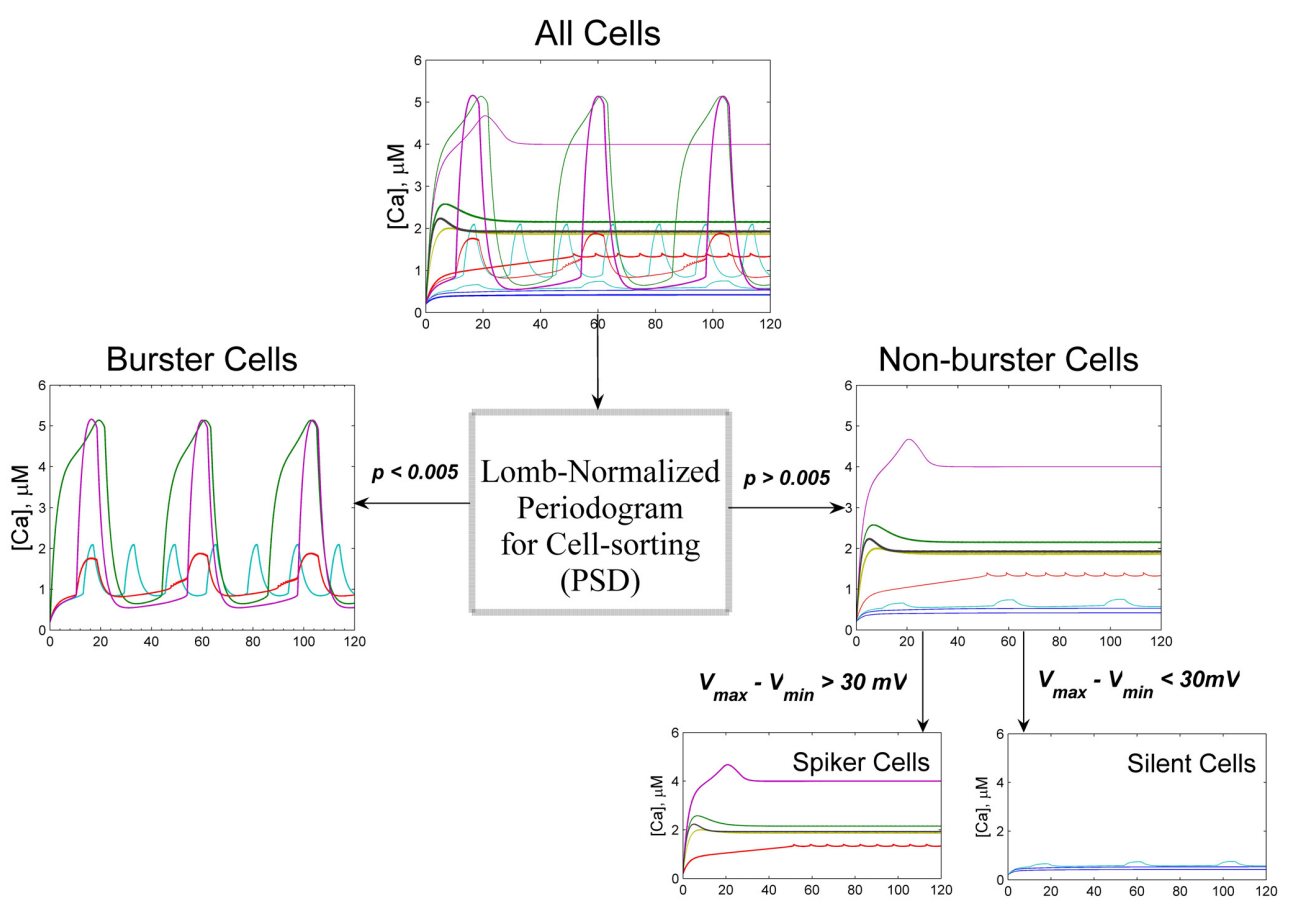
The flowchart of our cell sorting algorithm. Intra-cellular calcium signals, *Ca*(*t*) are passed to a program which calculates the periodogram PSD and the probability of the peak PSD values. If *p *< 0.005, cell is sorted as a burster cell. Among non-bursters, cells which satisfy the condition Δ*V *= |*V*_max _- *V*_min_| < 30 mV are considered silent cells, and the rest as spiker cells.

The low-limit of *f *is taken to be 1/*T*, where *T *is the time span and is equal to *t*_N _- *t*_1_. Since our simulations are carried out for a period of 120 sec, *f *is 0.0083 Hz. The uplimit of *f *is taken as the Nyquist frequency, *N*/(2*T*), where *N *is the length of the dataset. This gives a value of 1.0 Hz. Scargle showed that the null distribution of the Lomb-Scargle periodogram at a given frequency is exponentially distributed, namely the cumulative distribution function of *P*(*ω*) is given by Pr [*P*(*ω*) <*z*] = 1 - *e*^-*z *^[47]. Therefore, once *P*(*ω*) is calculated for different frequencies, the significance of the principal peak, *max*(*P*(*ω*)) can be evaluated by [47]:

(10) *p *= 1 - (1 - *e*^-max(*P*(*ω*))^)^*M*^

where *M *equals number of independent test frequencies. In our case it equals the number of data points *N*. Expression (10) tests the null hypothesis that the peak is due to random chance. When *p*-value of the principal peak is small, the time series is considered to contain significant periodic signal, and in our case, the cell can be considered a burster with regular oscillatory pattern. In this study the threshold *p*-value for burster cell is set to be 0.005. Among non-bursters, cells whose maximum and minimum membrane voltages differ by less than 30 mV, Δ*V *= |*V*_max _- *V*_min_| < 30 mV, are sorted as silent cells and the rest as spikers. The flowchart of the complete sorting process is presented in figure 6. For the burster *β*-cells, their bursting periods *T*_b _and degree of synchronization in bursting were then determined.

### Synchronization Analysis

Briefly, the instantaneous phase of each *β*-cell was first determined using the Matlab command: *φ*_*j*_(*t*) = unwrap(angle(Hilbert(detrend(*V*_*j*_(*t*)))). A mean field value of phase Φ is determined by taking the circular mean of the individual phase angles of all bursting *β*-cells

(11) Φ(*t*_*k*_) = arg ∑ exp (*iφ*_*j*_(*t*_*k*_))

The synchronization strength to mean field by each *β*-cell can be calculated by

(12) *ρ*_*j *_= |⟨ exp(*iφ*_*j*_(*t*_k_) - Φ(*t*_*k*_))⟩|

A cluster synchronization index (CSI) is then defined by

(13) $$\text{CSI}=\langle \rho_{j}\rangle =\frac{1}{n_{\beta }}\sum_{j}\rho_{j}$$

It measures how cells in the whole cluster are coupled in their oscillation. When synchronization is evaluated among bursting *β*-cells only, a simpler approach that measures the mean pair-wise phase difference can be taken. The synchronization of each pair of cells *j *and *k *is calculated by

(14) *λ*_*j,k *_= |⟨ exp(*i*(*φ*_*j*_(*t*) - *φ*_*k*_(*t*))⟩|

The mean of all pair-wise synchronization are then determined by:

(15) $$\lambda =\langle \lambda_{j,k}\rangle =\frac{2}{n_{\beta }\left(n_{\beta }-1\right)}\sum_{j,k>j}^{n_{\beta }}\lambda_{j,k}$$

For each cluster both the mean value and the distribution of CSI and *λ *are evaluated. The results are compared to reveal if there is modular pattern within the cluster, namely, if there are sub-regions within the whole cluster where the *β*-cells within each region is well synchronized, but not with *β*-cells in the other sub-regions. In the *β*-clusters we have simulated, the results of CSI and *λ *are not significantly different, and therefore for simplicity we only report the results of *λ*.

## Abbreviations

CCI: cluster coupling index; CSI: cluster synchronization index; HCP: hexagonal closest packing; SCP: simple cubic packing; T1D: type 1 diabetes; T2D: type 2 diabetes; ROI: region of interest; PSD: power spectral density.

## Competing interests

The authors declare that they have no competing interests.

## Authors' contributions

AN and XW both contributed to the development of the modeling method. AN wrote the Matlab code and ran the simulation. Both contributed to the writing of the manuscript, read and approved the final manuscript.
